# A Rapid Deployment Method for Real-Time Water Surface Elevation Measurement

**DOI:** 10.3390/s25061850

**Published:** 2025-03-17

**Authors:** Yun Jiang

**Affiliations:** College of Information Science and Engineering, Hohai University, Changzhou 213200, China; 221307020031@hhu.edu.cn

**Keywords:** elevation measurement, binocular stereo vision, stereo match, plane fitting

## Abstract

In this research, I introduce a water surface elevation measurement method that combines point cloud processing techniques and stereo vision cameras. While current vision-based water level measurement techniques focus on laboratory measurements or are based on auxiliary devices such as water rulers, I investigated the feasibility of measuring elevation based on images of the water surface. This research implements a monitoring system on-site, comprising a ZED 2i binocular camera (Stereolabs, San Francisco, CA, USA). First, the uncertainty of the camera is evaluated in a real measurement scenario. Then, the water surface images captured by the binocular camera are stereo matched to obtain parallax maps. Subsequently, the results of the binocular camera calibration are utilized to obtain the 3D point cloud coordinate values of the water surface image. Finally, the horizontal plane equation is solved by the RANSAC algorithm to finalize the height of the camera on the water surface. This approach is particularly significant as it offers a non-contact, shore-based solution that eliminates the need for physical water references, thereby enhancing the adaptability and efficiency of water level monitoring in challenging environments, such as remote or inaccessible areas. Within a measured elevation of 5 m, the water level measurement error is less than 2 cm.

## 1. Introduction

Water level measurement is fundamental to the hydrological monitoring of rivers, lakes, and reservoirs, and timely and accurate water level measurements are crucial for water resource management and the early warning and forecasting of water disasters [1]. In the field of hydrological monitoring, existing water level measurement techniques are primarily divided into two categories: contact and non-contact [2].

Contact water level meters primarily include the following: (1) float-type water level meters [3,4,5,6,7], which offer good stability and high reliability and are generally applicable within a 40 m range of water level variation, but require the use of logging wells, resulting in high construction, operation, and maintenance costs, while also being unsuitable for rivers with severe siltation or gently sloping cross-sections that present sizing problems; (2) pressure-type water level meters, which have a simple structure, are inexpensive, and are typically used within a 20 m range of water level variation; however, they are not suitable for water bodies with high sand content, nor are they suitable for estuaries and other areas where seawater is affected by changes in water density and severe siltation at measurement points; (3) liquid-mediated ultrasonic water level meters, which are ideal for measurement points without the option to construct water level wells. They usually require installation at least 0.5 m below the lowest water level and are unsuitable for silt channels. They also experience problems with temperature and time drift [4].

Non-contact water level meters primarily include the following. (1) Gas-mediated ultrasonic water level meters [5] have a simple principle and are cost-effective, suitable for installation at least 0.5 m above the highest water level. However, they require ensuring that the transducer’s beam angle is not obstructed within the reflector’s range and are not suitable for measurements on gentle slopes. Additionally, they experience more significant temperature and time drift issues. (2) Laser water level meters offer high ranging accuracy up to the millimeter level but require a water level logging well [5]. (3) Radar water level meters [8,9,10,11] have higher measurement accuracy, reaching the centimeter level, and exhibit no significant temperature or time drift issues. However, they must be installed vertically above the measured water body and are not suitable for measurements on gentle bank slopes. Additionally, the radar signal beam angle range must be unobstructed, and rain or snow may interrupt the measurement [6]. (4) Visual water level meters primarily function by recognizing the water body in the water ruler and other references for water level line readings to convert water levels. In principle, they exhibit no temperature or time drift, and can perform tilt detection, providing intuitive results that facilitate visual calibration. However, this method is highly sensitive to complex lighting conditions (such as reflection and flare) [12], adverse weather (like fog and heavy rain), and water flow disturbances (including waves and floating objects) on the water surface. Entanglement of the water ruler by floating objects is also a significant concern, as it can lead to measurement failure or even damage to the water ruler, requiring manual intervention for maintenance. Consequently, visual water level meter implementation is typically limited to scenarios with vertical slopes or bridge abutments, as well as other stable installation settings.

Binocular stereo vision technology [13], known for its contactless and simple structure, is widely used in fields such as industrial precision measurement, autonomous driving, robot navigation, and medical diagnosis [14,15,16], making it one of the most prevalent methods for three-dimensional information perception. Binocular stereo vision places two vision sensors at different positions and observes the same scene from different perspectives at the same time to obtain a set of stereo image pairs containing scene depth information and then calculates depth information using an appropriate algorithm.

In stereo vision applications, the baseline length of the binocular camera directly influences the measurement range and accuracy [17]; thus, the appropriate baseline length should be determined based on the camera’s focal length and the actual detection distance. The technical core of the water level measurement method based on binocular stereo vision is obtaining accurate parallax maps from the left and right eye images and using the parallax principle to obtain 3D point cloud data. Currently, parallax map acquisition methods can be divided into two categories: traditional stereo matching and deep learning stereo matching. Among these, traditional stereo matching algorithms include BM (block matching) [18], SGBM (semi-global block matching) [19], Census [20], and others. Water surface images are prone to specular reflection, shadows, flare, ripples, and other optical noise, complicating feature extraction and matching in areas with weak and repeated textures. Moreover, bank slopes, vegetation, and floating objects can obstruct the water surface, leading to voids in the parallax map or inaccuracies in representing real water surface anomalies. Consequently, using the parallax map to obtain three-dimensional point cloud data and performing point cloud plane fitting are essential steps to acquire the water level value. Classical plane fitting methods [21,22,23,24] include the least squares methods and eigenvalue methods, among others. However, these methods cannot eliminate the influence of outliers on plane fitting, leading to low accuracy and poor robustness in horizontal plane fitting. Deep learning methods can extract complex global features and contextual information and offer more significant advantages in handling water surface environments with specular reflection, weakly textured, or even untextured regions. However, no high-precision parallax map datasets are available for real water surface environments in the stereo vision research field [25,26,27,28]. Consequently, deep learning stereo matching models trained directly on existing terrestrial and synthetic datasets often lack sufficient generalization ability.

The proposed method of using binocular stereo vision for water surface height measurement represents a significant advancement compared to existing techniques. Unlike traditional contact methods, which require physical contact with the water and are susceptible to environmental interference, our approach is entirely non-contact. This eliminates the need for costly and complex infrastructure such as logging wells, making it more practical for a wide range of hydrological monitoring scenarios. Additionally, compared to other non-contact methods, binocular stereo vision offers several unique advantages. For instance, it does not require the installation of additional reference markers or devices, such as water rulers or laser targets, which are often necessary for visual water level meters or laser-based systems. This reduces the complexity and potential for errors associated with these methods.

In this context, this study examines the measurement accuracy of the binocular system using the binocular stereo vision method, tailored to the actual measurement scenarios. Subsequently, a sequence of water surface images is synchronously captured in grayscale using binocular cameras, and stereo matching algorithms are employed to obtain the parallax values of the left and right images, thereby reconstructing a three-dimensional water surface point cloud. The water surface point cloud was fitted to a plane to obtain elevation values from the camera to the fitted horizontal plane, and the acquired elevation data were verified against laser rangefinder measurements.

## 2. Materials and Methods

**Figure 1:**
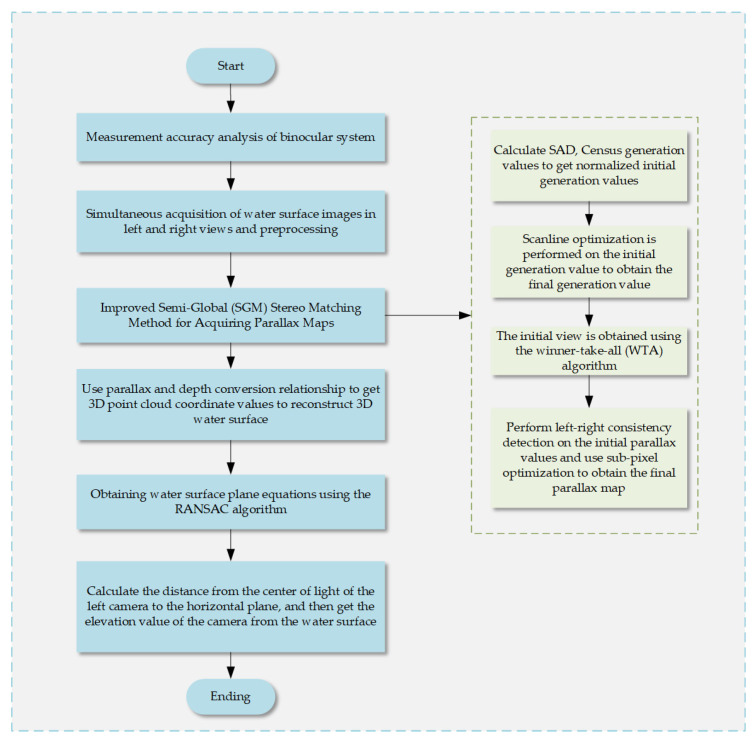
Elevation measurement system realization flow chart.

The implementation process of the water level measurement system, depicted in Figure 1, comprises five steps: selecting the binocular camera, simultaneously acquiring and preprocessing water surface images, performing stereo matching, obtaining 3D point cloud coordinate values, fitting a horizontal plane, and acquiring elevation values. First, the stereo-corrected initial image of the water surface, synchronously acquired by the binocular camera, is converted to grayscale and preprocessed. Then, the SAD-Census algorithm calculates the cost on a pixel-by-pixel basis, and an iterative SGM (semi-global matching) algorithm is employed for matching cost aggregation to generate the parallax map. Next, based on the binocular camera’s calibration results, the relationship between parallax and depth is used to obtain the 3D point cloud coordinate values of the water surface image. The point cloud is fitted to a plane using the RANSAC (random sample consensus) algorithm [29,30,31] to generate the horizontal plane equations. Finally, the distance from the optical center of the left camera to the horizontal plane is calculated to obtain the elevation value of the camera from the fitted horizontal plane.

### 2.1. Principle of Binocular Vision

When a spatial point is projected onto the two cameras of the binocular vision system via pinhole imaging, the two image points and the target point form a triangle. Using the parallax principle and the camera’s internal and external parameters, the 3D coordinates are determined. This process employs the principle of triangular similarity, thereby obtaining the spatial position information of the point. Assuming that the spatial target point has the coordinates in the pixel coordinate system as (u,v), the coordinates under the image coordinate system as (x,y), the coordinates in the camera coordinate system as $\left(X_{c},Y_{c},Z_{c}\right)$, and the coordinates in the world coordinate system as $\left(X_{w},Y_{w},Z_{w}\right)$, this is obtained according to the pinhole camera imaging principle [32]:

(1) $$Z_{c}\left[\begin{array}{c} u\\ v\\ 1 \end{array}\right]=\left[\begin{array}{cccc} f_{x} & 0 & c_{x} & 0\\ 0 & f_{y} & c_{y} & 0\\ 0 & 0 & 1 & 0 \end{array}\right]\left[\begin{array}{c} X_{c}\\ Y_{c}\\ Z_{c}\\ 1 \end{array}\right]=\left[\begin{array}{cc} K & 0 \end{array}\right]\left[\begin{array}{cc} R & T\\ 0 & 1 \end{array}\right]\left[\begin{array}{c} X_{w}\\ Y_{w}\\ Z_{w}\\ 1 \end{array}\right],$$

where $f_{x}$ and $f_{y}$ are the focal lengths of the camera (in pixels) along and in the direction of the image; $c_{x}$ and $c_{y}$ are the coordinates of the principal point (the center of light of the image); (x,y,1) is the chi-square coordinate of the measurement point in the image coordinate system; $\left(X_{c},Y_{c},Z_{c},1\right)$ is the chi-square coordinate of the measurement point in the camera coordinate system; K is the camera’s internal reference matrix; and R and T denote the rotation and translation matrices between the camera coordinate system and the world coordinate system, respectively, with the origin of the world coordinate system being the left camera’s optical center.

Based on the principle of triangulation [33], the expression for depth $Z_{c}$ can be deduced as the following:

(2) $$Z_{c}=\frac{f\cdot B}{u_{r}-u_{l}}=\frac{f\cdot B}{d},$$

where $u_{l}$ and $u_{r}$ are the coordinates of the intersection of the measurement point P and the imaging plane in the direction u, and d is the difference in u-direction coordinates of the point of the same name, also known as parallax.

### 2.2. Measurement Accuracy Analysis of Binocular System

**Figure 2:**
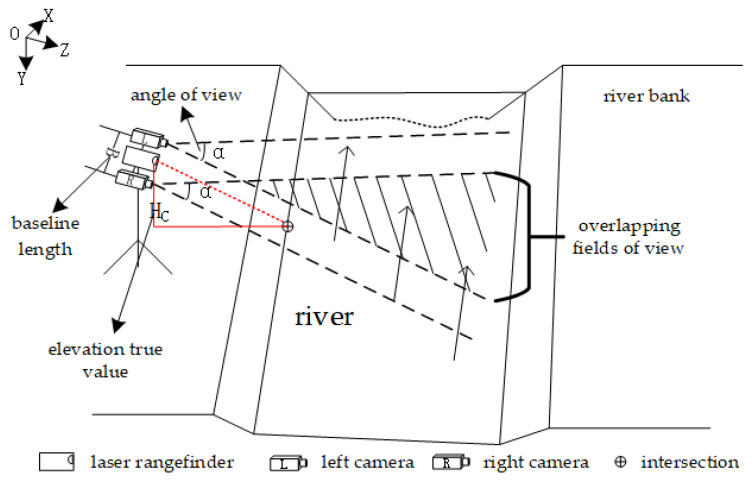
Principle of water surface elevation calculation by binocular stereo vision.

This study examines the accuracy of binocular stereo vision in real-world measurement scenarios. To ensure that the common field of view of the binocular camera covers at least two thirds of the field of view of either the left or right eye during measurement, I analyzed how the field of view angle and baseline length of the binocular camera affected the depth measurement distance, as per Equation (2). Equation (3) is derived from Figure 2:

(3) $$\frac{Z}{\tan\frac{\alpha }{2}}\sin\frac{\alpha }{2}-\frac{B}{2}\geq \frac{2}{3}\cdot \frac{B}{2}\;\Rightarrow \frac{4B}{3\cos\frac{\alpha }{2}}\leq Z,$$

For $Z_{c}$, d is the independent variable and a partial derivative is applied to d:

(4) $$\frac{\partial Z}{\partial d}=\frac{-Bf}{d^{2}}=\frac{-Z^{2}}{Bf}$$

By multiplying Equation (4) by the parallax accuracy $s_{d}$, I derived Equation (5). It is evident that the focal length f, baseline length B, and measurement distance Z significantly impact system accuracy $s_{z}$, particularly when the parallax accuracy $s_{d}$ is one pixel.

(5) $$s_{z}=\frac{Z^{2}}{Bf}\cdot s_{d}$$

### 2.3. Parallax Map Acquisition Methods

In this study, I employ an iterative optimization-based SGM stereo matching algorithm, considering that the matching cost at a single pixel is often influenced by image noise, uneven illumination, and other factors. Initially, I use the SAD and Census algorithms to compute the initial proxy value for each pixel in the water surface image, leveraging neighborhood pixel information to mitigate brightness anomalies caused by water surface reflection. Then, an iterative scanline optimization algorithm optimizes the aggregation of the initial generation values, and for each pixel, the parallax value corresponding to the smallest optimized generation value is selected to form the initial parallax map. Finally, leveraging the parallax consistency constraints, the initial parallax values are optimized through consistency detection and sub-pixel optimization methods to generate the final parallax map.

#### 2.3.1. Calculation of Consideration

The fundamental principle of the SAD algorithm involves using a fixed window size. This window moves horizontally from left to right across the image, calculating the absolute difference of the gray values between corresponding pixels in the left and right images within this window. The SAD cost calculation function is expressed as the following:

(6) $$C_{SAD}(p,d)=\sum_{-h}^{h}\sum_{-w}^{w}\left|I_{L}(x+i,y+j)-I_{R}(x+d+i,y+j)\right|,\;\;\;\;\;\;d\in [d_{\min},d_{\max}],$$

where $I_{L}$ and $I_{R}$ are the grayscale values of the left and right views; d is any value between the maximum parallax $d_{\max}$ and the minimum parallax $d_{\min}$; and h and w are the length and width of the computational window.

The Census transform generates a bit string by comparing the gray value of the center pixel with those of the surrounding pixels, and uses the comparison result in the following formula:

(7) $$\left\{\begin{array}{l} C_{s}(p)=⊗\xi (I(p),I(q)),q\in N_{p}\\ \xi (I(p),I(q))=\left\{\begin{array}{l} 0,\;I(p)\leq I(q)\\ 1,\;I(p)>I(q) \end{array}\right. \end{array}\right.,$$

where I(p), I(q) are the gray values at pixel points p, q; ⊗ denotes a bitwise connection; $N_{p}$ denotes the neighborhood of the pixel p; and ξ(I(p),I(q)) is the binary code obtained by comparing the size relationship at any point of the left view. Based on the above equation, a bit string is obtained, and the Hamming distance between the bit strings of corresponding pixels in the left and right views is calculated to obtain the surrogate value, and the Census cost calculation function is derived:

(8) $$C_{Census}(p,d)=\mathrm{Hamming}(C_{ls}(p),C_{rs}(p-d)),\;\;\;\;\;\;d\in [d_{\min},d_{\max}],$$

where $C_{ls}(p)$ is the bit string corresponding to the pixel point in the left view; $C_{rs}(p-d)$ is the bit string corresponding to the pix point in the right view; and $C_{Census}(p,d)$ is the Hamming distance.

The SAD and Census transformation algorithms are normalized to obtain the initial proxy value, and the function is calculated as shown in Equation (9):

(9) $$C(p,d)=\rho (C_{Census}(p,d),\lambda_{Census})+\;\rho (C_{SAD}(p,d),\lambda_{SAD}),d\in [d_{\min},\;d_{\max}],$$

#### 2.3.2. Cost Aggregation

After obtaining the initial surrogate value, it is propagated from the high SNR to the low SNR region by multipath cost aggregation, and the surrogate value of the weak texture region is obtained by extrapolating the surrogate value from the rich texture region based on the smoothness and continuity of the water surface to obtain the optimized surrogate value of the left and right water surface images, which improves the matching accuracy of the weak texture region. In this paper, I use 8-path cost aggregation with multiple iterations, and the calculation formula is as follows:

(10) $$C_{r}(p,d)=C_{1}(p,d)+\min\left\{\begin{array}{l} C_{r}(p-r,d),\\ C_{r}(p-r,d\pm 1)+P_{1}\\ \underset{k}{\min}C_{r}(p-r,k)+P_{2} \end{array}\right.-\underset{k}{\min}C_{r}(p-r,k),$$

where $C_{1}(p,d)$ is the algebraic value of the pixel at parallax. The second item is a smoothing item; $C_{r}(p-r,d)$ refers to the value of the aggregation generation for one pixel point along the direction of aggregation. $C_{r}(p-r,d\pm 1)$ refers to the value of aggregated surrogates that differ in parallax value along the direction of aggregation within one pixel of each other, and $\underset{k}{\min}C_{r}(p-r,k)$ refers to the minimum of all surrogate values where the parallax values differ by more than one pixel. The third term, $C_{r}(p,d)\leq C_{\max}+P_{2}$, ensures that the new path generation value does not exceed a certain value. Ultimately, the aggregated surrogate value $C_{agg}(p,d)$ of pixel p at parallax d is as follows:

(11) $$C_{agg}(p,d)=\frac{1}{8}\sum_{k}C_{r}(p,d)$$

#### 2.3.3. Parallax Calculation and Optimization

**Figure 3:**
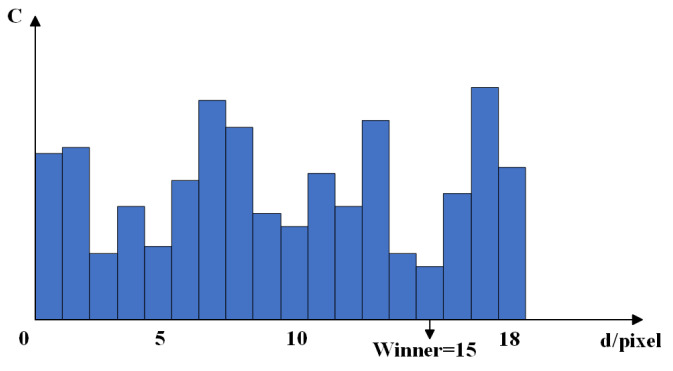
Schematic of winner-take-all algorithm.

Parallax calculations for the aggregated proxy values are performed using the winner-take-all (WTA) algorithm. That is, for each pixel, the parallax value corresponding to the smallest proxy value is selected as the initial parallax. The algorithm is schematically illustrated in Figure 3.

**Figure 4:**
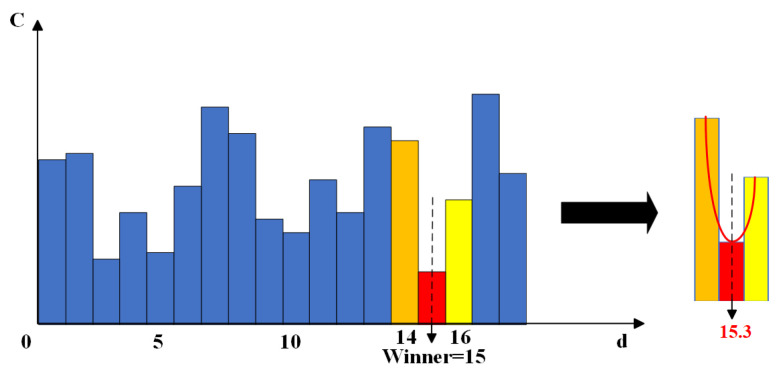
Schematic diagram of sub-pixel optimization algorithm.

To mitigate the high false matching rate attributed to brightness disparities between the left and right images, I applied the parallax consistency constraint, conducted a consistency test on the initial parallax map, and eliminated invalid pixel points. Following the consistency check, the sub-pixel optimization technique was employed to enhance parallax accuracy. This technique further refines the pixel-level divisions by forming a parabola using the optimal parallax value and its adjacent left and right parallax values, substituting a very small value for the previously calculated optimal value to achieve sub-pixel level parallax optimization. The calculation function is presented in Equation (12). The principle is illustrated in Figure 4.

(12) $${D^{*}}_{p}=D_{p}-\frac{C_{agg}(p,d+1)-C_{agg}(p,d-1)}{2(C_{agg}(p,d+1)-2C_{agg}(p,d)+C_{agg}(p,d-1))},$$

where ${D^{*}}_{p}$ is the optimized parallax value; $D_{p}$ is the parallax value of the pixel p; and $C_{agg}(p,d+1)$ and $C_{agg}(p,d-1)$ are the aggregation costs of p at the parallaxes of d+1 and d−1, respectively.

### 2.4. Methods for Obtaining Water Level Values

Utilizing the dense parallax map obtained, I initially acquired the 3D point cloud coordinates for each pixel in the water surface image using Equation (2) to reconstruct the water surface in three dimensions. Subsequently, employing the RANSAC algorithm, I processed the 3D point cloud to fit a plane and generated the equation of the horizontal plane. Lastly, I calculated the distance from the optical center of the left camera to the horizontal plane to determine the elevation of the water surface relative to the binocular camera.

The fundamental principle of the RANSAC algorithm for fitting a horizontal surface model is as follows:

(1) Three randomly selected non-collinear points from the water surface 3D point cloud coordinate dataset are used to determine preliminary planar model Ax+By+Cz+D=0, where, A, B, C, D are the coefficients of the model;
(2) The distance h of every other data point $s_{j}\in S$ to this plane is calculated according to Equation (13); threshold τ is set. If h≤τ, then $s_{j}$ denotes the interior point and the number of interior points is denoted as $M_{i}$;
  (13) $$h=\frac{\left|Ax_{0}+By_{0}+Cz_{0}+D\right|}{\sqrt{A^{2}+B^{2}+C^{2}}},$$
  (14) $$M_{i}=\sum_{j=1}^{n}I(s_{j}\leq \tau ),$$
  where I is an indicator function that takes the value 1 when condition $s_{j}\leq \tau$ holds and 0 otherwise and is the total number of data in the dataset, and n is the total number of data in dataset S;
(3) According to Equation (14), if the number of interior points ${M_{i}}^{\prime }$ of the current model exceeds the previous maximum number of interior points $M_{i}$, then the optimal model parameters are updated to $A^{\prime }x+B^{\prime }y+C^{\prime }z+D^{\prime }=0$;
(4) The above steps are repeated until a preset number of iterations (1000) is reached, and the model with the highest number of interior points is selected as the final result.

The camera elevation value H from the water surface is calculated as follows:

(15) $$H=\frac{\left|D\right|}{\sqrt{A^{2}+B^{2}+C^{2}}}$$

## 3. Results

Given the complex lighting effects in the field measurement environment, this condition is not conducive to water surface imaging and the synchronous capture of high-resolution water surface images. To address this, the study employs the ZED 2i binocular camera as the experimental equipment. The camera’s polarizer configuration reduces water surface reflections, thereby improving image clarity and contrast, which facilitates subsequent image processing. The specific parameters are shown in Table 1.

### 3.1. Measuring Range of Binocular System

**Figure 5:**
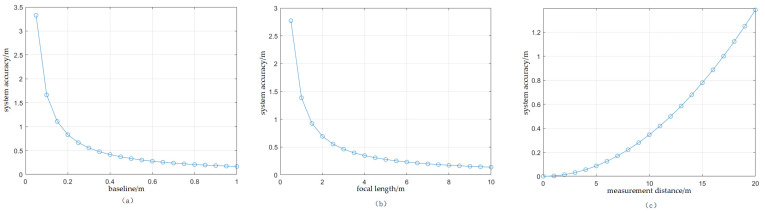
Accuracy analysis result graph. (**a**) Effect of baseline; (**b**) effect of focal length; (**c**) effect of measurement distance.

This study analyzes the factors influencing the accuracy of the system using the ZED 2i binocular camera. According to Equation (5), the parameters that affect the accuracy of the binocular system include the camera baseline length B, the camera focal length f, and the measurement distance Z. The parameters are set as shown in Table 1. The effect of each of the three parameters on the system’s accuracy is analyzed, and the results are shown in Figure 5. It is observed that when the measurement distance is fixed, the longer the baseline and the greater the focal length, the higher the system accuracy; when the baseline and the focal length are fixed, the greater the measurement distance, the lower the system accuracy.

As can be seen from Formula (3), under the current binocular camera parameter settings at the depth measurement distance Z≥0.129 m, as illustrated in Figure 5c, to achieve a relative error of less than 3%, the measurement distance should be less than 10 m. In summary, when the binocular camera has a baseline of 120 mm and a field of view of 72°, the measurement distance range to meet actual measurement needs is 0.129 m≤Z≤10 m.

### 3.2. Camera Parameter Calibration

The binocular camera must be calibrated before the 3D reconstruction of the water surface. Using Zhang Zhengyou’s calibration method [34], the internal reference matrices $M_{L}$ and $M_{R}$, rotation matrix R, and translation vector T for both the left and right cameras were obtained, as shown in Table 2.

### 3.3. Experimental Setup

The experimental sites were located at Dongshan Station on the Qinhuai River in Nanjing, Jiangsu Province, and at East Lake on the Jiangning Campus of Hohai University. Dongshan Station, on the Qinhuai River, is located in a critical hydrological monitoring area of the Qinhuai River Basin, and its data are essential for flood control and water resource management. The East Lake provides a controlled environment with stable water levels and diverse surrounding conditions, including vegetation and partial obstructions. Thus, these are ideal locations to test the robustness and accuracy of my water level measurement method under real-world river conditions. Water surface images were collected in three different scenarios. The resolution of the images was uniformly set to 1920 × 1080 pixels for research and comparison purposes.

**Figure 6:**
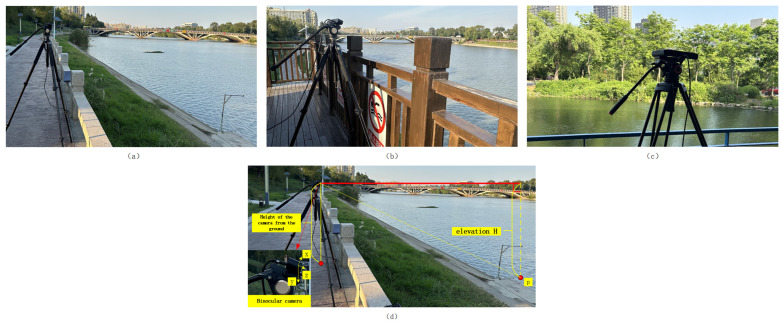
The site layout plan. (**a**) The riverbank at Dongshan Station; (**b**) the observatory at Dongshan Station; (**c**) East Lake of Hohai University; (**d**) the overall camera setup diagram.

**Figure 7:**
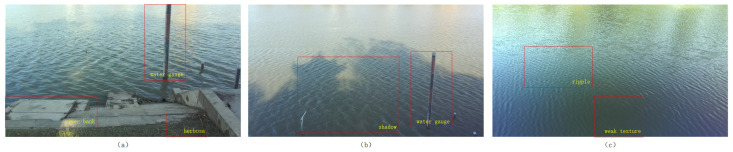
Water surface image dataset. (**a**) Contains water gauge, riverbanks, and grass; (**b**) contains water gauge and shadows; (**c**) contains ripples and weak texture.

The on-site system deployment is shown in Figure 6d. Figure 6a shows the camera position erected on the riverbank at Dongshan Station, and the captured water surface images correspond to Figure 7a. Figure 6b shows the camera position on the observatory at Dongshan Station, and the captured water surface images correspond to Figure 7b. Figure 6c shows the camera position at East Lake of Hohai University, and the captured water surface images correspond to Figure 7c. The pitch angle is adjusted during the deployment of the binocular camera. This ensures that the left and right views cover the entire water surface area across the full range of water levels, and synchronized acquisition of the water surface images from both views is utilized. The corresponding three scenarios in Figure 7 are as follows: Figure 7a contains noise sources such as water gauges, riverbanks, and grass; Figure 7b contains noise sources such as large shadows and water gauges; Figure 7c contains water surface ripples as well as weakly textured areas.

### 3.4. Water Surface Parallax Map Results

**Figure 8:**
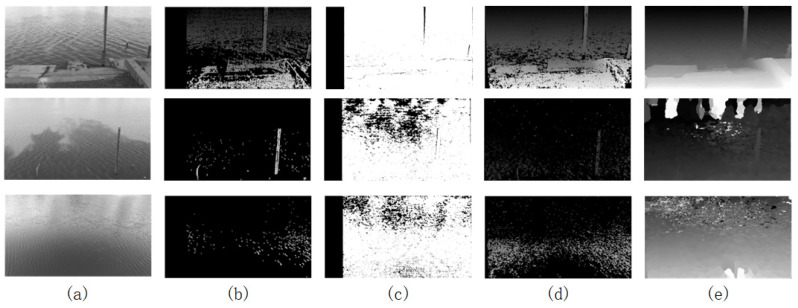
Parallax maps generated by different algorithms. (**a**) Original grayscale image; (**b**) BM; (**c**) SGBM; (**d**) SGM; (**e**) algorithms in this paper.

To verify the feasibility and generalizability of the improved SGM algorithm, the improved SGM algorithm is compared with the classical algorithms BM, SGBM, and SGM. The water surface parallax generated by these four algorithms is shown in Figure 8.

### 3.5. Elevation Measurement Results

**Figure 9:**
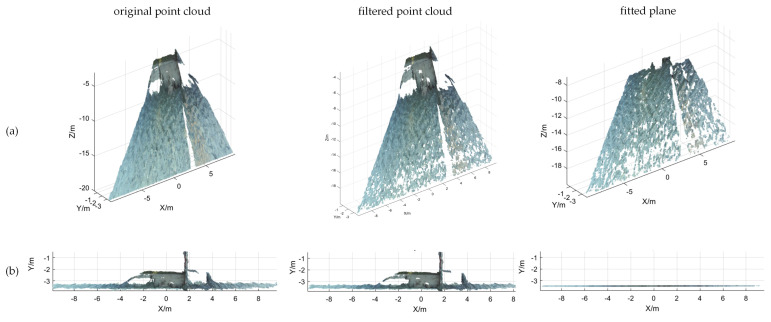
Point cloud processing result map. (**a**) 3D view; (**b**) XY-axis view.

**Figure 10:**
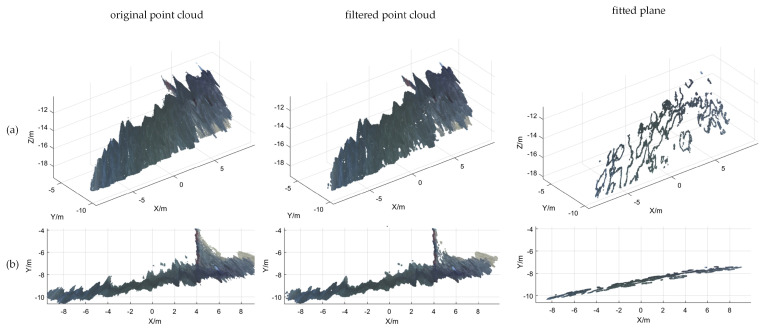
Point cloud processing result map. (**a**) 3D view; (**b**) XY-axis view.

**Figure 11:**
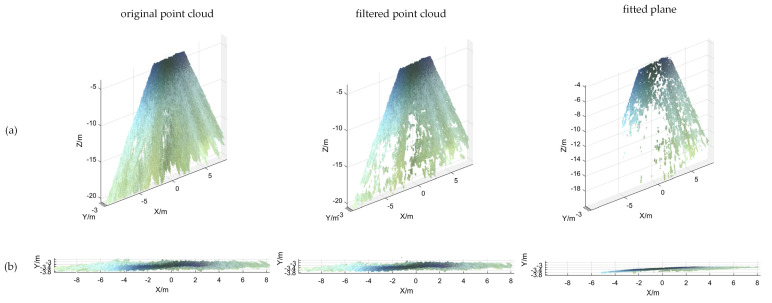
Point cloud processing result map. (**a**) 3D view; (**b**) XY-axis view.

Based on the calibration results of the binocular camera, the 3D point cloud reconstruction of the water surface was conducted, and the results are shown in Figure 9, Figure 10 and Figure 11. In Figure 9a, Figure 10a and Figure 11a, the point cloud processing for the 3D view can be seen, which includes filtering as well as plane fitting. To make it easier to read, the article also adds a point cloud process for the XY-axis view, as shown in Figure 9b, Figure 10b and Figure 11b. The equations of the horizontal plane and the measurement results of the water level values are shown in Table 3. The algorithm can fit an effective horizontal plane in complex scenes. The fitting results not only accurately reflect the water surface position, but also resist anomalies caused by water surface fluctuations or other factors in complex scenes, demonstrating high fitting accuracy and generalizability.

**Figure 12:**
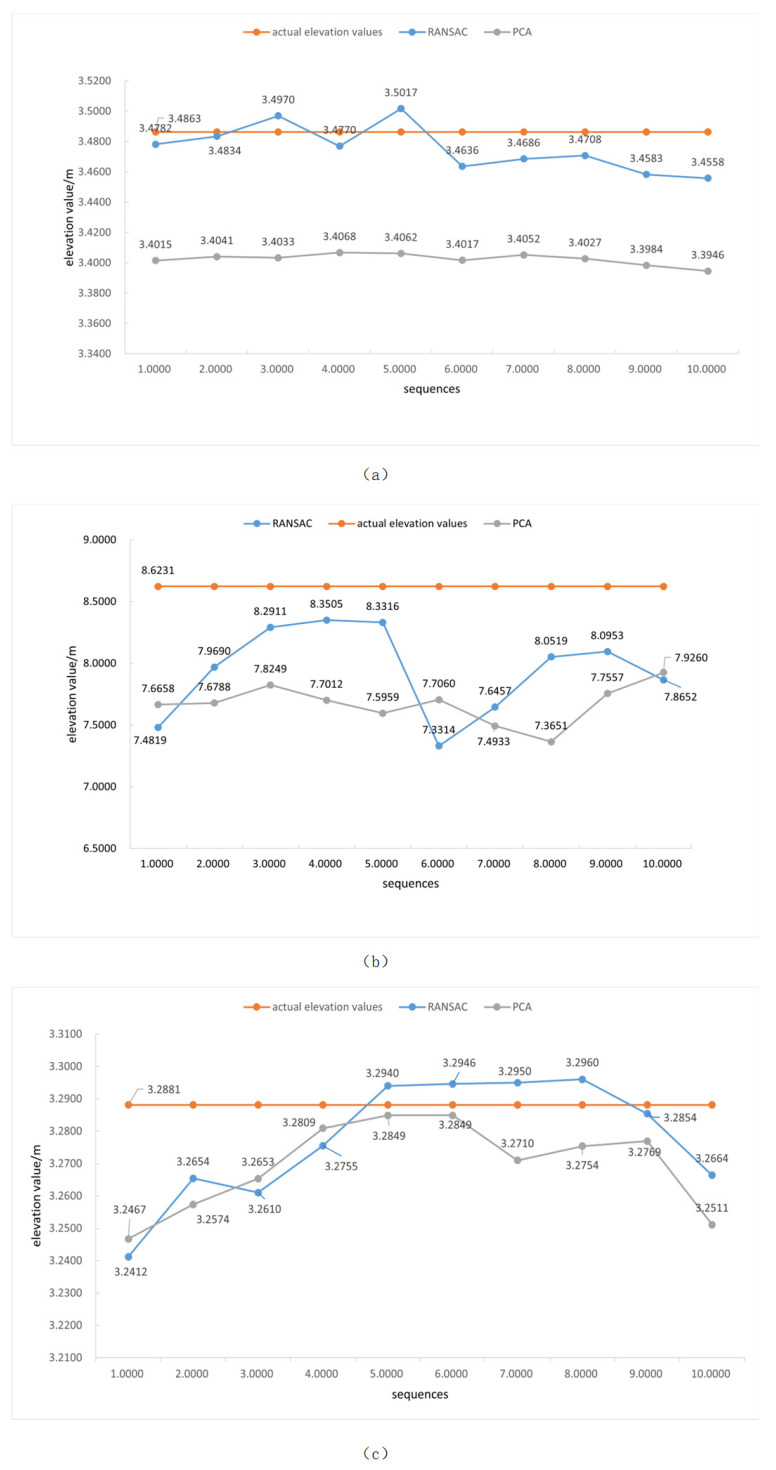
Elevation measurements and comparative experimental results. (**a**) Scene A; (**b**) Scene B; (**c**) Scene C.

To quantify the computational accuracy of the measurement algorithm presented herein, the elevation values were calculated using 10 sets of image data corresponding to the three aforementioned scenarios, and the results are presented in Figure 12. The computational time for stereo matching is shown in Table 4. The Mean Absolute Error (MAE) and Root Mean Square Error (RMSE) of the elevation values obtained from the measurements and the camera elevation obtained from the laser rangefinder are used as the evaluation criteria for the algorithm presented herein. In scenarios (a), (b), and (c), the MAE of the elevation values computed by the method presented in this paper are 1.6 cm, 68.1 cm, and 1.6 cm, and the RMSE values are 1.8 cm, 76.1 cm, and 2.1 cm, respectively.

Meanwhile, in order to find a more suitable plane-fitting method, I have included a comparison with the PCA (Principal Component Analysis) method in the experimental section. The results are presented in Figure 12. From the experimental results, it can be seen that in scenario (a), the PCA method is less effective than the RANSAC algorithm, with an MAE of 8.4 cm. In scenario (b), although the RMSE of 11.3 cm is more stable than that of the RANSAC algorithm, the MAE of 95.2 is far away from the true value. In scenario (c), the MSE and the RMSE are 1.9 cm and 1.2 cm, and the algorithm has a worse effect as a whole, which verifies the effectiveness of RANSAC in this paper.

## 4. Discussion

From Figure 8, it is evident that the BM and SGBM algorithms obtain less effective information in environments affected by water surface shadows, and the generated parallax maps are more sparse and can only reflect the contour of the water ruler. The parallax map provides richer depth information in strongly textured scenes, such as riverbanks. However, it still contains more holes in the water surface area and fails to reflect the structural characteristics of the water surface. The water surface parallax map generated by the traditional SGM algorithm recovers clearer water surface ripples in regions with obvious water surface texture compared to the previous two algorithms. However, it still struggles to handle large areas with weak texture effectively. The algorithm proposed herein acquires more effective information.

As shown in Figure 8e, in the first scene, the water scale and near shore show clearer water surface ripples compared to the previous three methods, which lack obvious depth information. In the second scenario, the algorithm presented herein obtains an extremely dense parallax map, with obvious water surface ripples and rich depth information of the riverbank, demonstrating its effectiveness. The third scenario obtained more water surface information compared to the first three methods but still exhibited many holes, with no depth information for the far shore and highly reflective water surface. This is achieved through enhanced cost calculation methods and the iterative optimization of cost aggregation, thereby obtaining relatively clear water surface structural features in complex water surface scenes while still achieving a more accurate parallax value in large, texture-free areas. Moreover, compared to traditional methods, the algorithm presented herein only takes longer than BM in terms of execution time. In all three scenarios, the algorithm demonstrates significant real-time performance advantages.

The maximum effective distance of the ZED 2i binocular camera used in this study for water level measurement is primarily constrained by the camera’s parallax range and measurement accuracy. In my experiments, the measurement error of this method was found to be less than 2 cm within a 5 m elevation range. However, as the distance increases, the parallax value decreases, leading to reduced measurement accuracy. In practice, it is recommended to use this method within 5 m to ensure high measurement accuracy. The baseline distance of the stereo camera (i.e., the distance between the two camera lenses) has a significant effect on measurement accuracy. Longer baseline distances enhance parallax resolution, thereby improving measurement accuracy, but they also increase the risk of occlusion. In this study, the ZED 2i camera features a baseline distance of 12 cm, which has been experimentally verified to provide a good balance between accuracy and occlusion within the current measurement range. If longer distances need to be measured or higher accuracy is required, a binocular camera with a larger baseline distance should be considered, while the matching algorithm must be optimized simultaneously to minimize errors caused by occlusions.

Highly reflective or uniform water surfaces, such as calm water, pose a challenge for binocular stereo vision measurements. As these surfaces lack sufficient textural features, stereo matching becomes significantly more challenging. Although grayscale and gradient information were combined to improve matching accuracy in this study, and the images were captured under uniform lighting conditions whenever possible, significant challenges remain in extreme conditions, such as very calm water.

## 5. Conclusions

A shore-based water level measurement system was constructed using the principles of binocular stereo vision and point cloud processing. First, the binocular camera was selected. It was used to synchronously acquire the left and right eye water surface images. Then, the iterative SGM stereo matching algorithm was applied to obtain the water surface parallax map. Finally, the water level value was determined based on the reconstructed water surface three-dimensional data. The feasibility of the method for water level measurement was verified by analyzing the measurement results.

With the advancement of smart water resources and applications of binocular stereo vision, the following directions can be considered for future research to further enhance measurement accuracy: first, optimizing the stereo matching algorithm to improve matching accuracy under varying texture conditions; next, adjusting camera parameters, such as baseline distance and focal length, to accommodate measurements at different distances; additionally, exploring the use of environmental features, such as rippling water, to enhance the three-dimensional matching effect; and finally, investigating how to minimize the effects of viewing angle and lighting conditions on stereo matching accuracy during remote measurement. Through the combined application of these methods, it is anticipated that more accurate 3D measurement techniques will be developed in the future.

## Figures and Tables

**Table 1:** ZED 2i camera parameter list.

Specifications	ZED 2i
Resolution	2208 × 1242, 1920 × 1080, 1280 × 720, 662 × 376
Capture rate	up to100 FPS
Focal Length	4 mm
Field of View	72°
Baseline	120 mm
Depth Range	1.5–35 m
Depth Accuracy	<2% up to 10 m, <7% up to 30 m
Polarizing Filters	yes

**Table 2:** Camera calibration parameters.

internal reference matrix	$M_{L}=\left[\begin{array}{ccc} 1909.6100 & 0 & 986.5900\\ 0 & 1910.9100 & 526.0920\\ 0 & 0 & 1 \end{array}\right]$
	$M_{R}=\left[\begin{array}{ccc} 1909.2800 & 0 & 964.2200\\ 0 & 1909.8199 & 572.9490\\ 0 & 0 & 1 \end{array}\right]$
rotation matrix	$R=\left[\begin{array}{ccc} 0.9992 & 0.0013 & -0.0017\\ 0.0011 & 0.9999 & 0.0087\\ 0.0019 & -0.0085 & 0.9995 \end{array}\right]$
translation vector	$T=\left[\begin{array}{ccc} 120.1110 & -0.3757 & 0.4069 \end{array}\right]$

**Table 3:** Horizontal plane fitting parameters and elevation value measurements.

Scenes	A	B	C	D	Measured Elevation Values (m)
(a)	−0.001	1.001	−0.014	3.479	3.4770
(b)	0.163	0.980	−0.062	7.810	7.8069
(c)	0.002	1.001	−0.011	3.276	3.2755

**Table 4:** The computational time for stereo matching.

Sequences	1	2	3	4	5	6	7	8	9	10	Average Mean
**scenario (a)** **/s**											
BM	4.583	4.627	4.597	4.704	4.859	4.545	4.264	4.265	4.399	4.684	4.553
SGM	38.582	41.203	38.010	40.756	37.771	56.155	40.186	39.415	36.775	38.665	40.752
SGBM	82.279	82.338	81.857	82.422	81.148	84.002	81.323	80.090	81.394	85.244	82.210
this paper	32.218	32.345	37.678	31.987	35.432	38.123	33.789	36.543	32.001	34.872	**34.499**
**scenario (b)** **/s**											
BM	5.281	5.428	5.548	5.184	5.608	5.452	5.351	5.490	5.352	5.325	5.402
SGM	45.493	40.038	41.108	40.400	40.592	37.655	38.925	39.009	40.018	41.040	40.428
SGBM	85.641	84.568	84.116	83.670	84.923	108.230	83.089	83.747	83.941	83.704	86.563
this paper	35.123	34.765	37.321	38.210	35.123	39.543	40.652	39.543	32.876	40.256	**37.341**
**scenario (c)** **/s**											
BM	4.545	4.327	4.499	4.596	4.778	5.655	5.568	5.331	5.691	5.342	5.033
SGM	40.389	38.805	41.099	40.525	54.763	35.984	38.762	39.505	39.650	40.216	40.970
SGBM	83.005	82.080	81.732	81.807	82.659	83.280	85.576	85.494	81.786	85.492	83.291
this paper	35.126	36.789	37.169	37.522	33.610	34.358	37.572	36.969	35.163	36.882	**36.116**

## Data Availability

The data presented in this study are available on request from the author.

## References

[1] Liu J., Bao Z., Liu C., Wang G., Liu Y., Wang J., Guan X. (2019). Change law and cause analysis of water resources and water consumption in China in past 20 years. Hydro-Sci. Eng..

[2] Huang J., Zhang Y., Bing H., Peng J., Dong F., Gao J., Arhonditsis G.B. (2021). Characterizing the river water quality in China: Recent progress and on-going challenges. Water Res..

[3] Loizou K., Koutroulis E. (2016). Water level sensing: State of the art review and performance evaluation of a low-cost measurement system. Measurement.

[4] Djalilov A., Sobirov E., Nazarov O., Urolov S., Gayipov I. (2023). Study on automatic water level detection process using ultrasonic sensor. IOP Conf. Ser. Earth Environ. Sci..

[5] Masoudimoghaddam M., Yazdi J., Shahsavandi M. (2025). A low-cost ultrasonic sensor for online monitoring of water levels in rivers and channels. Flow Meas. Instrum..

[6] Liao A., Liu J., Zhang J., Jiang G., Zheng J., Wang N. (2019). Intercomparison of high-accuracy water level gauges in the scale of small experimental catchment. Adv. Water Sci..

[7] Ogasawara T., Ashida K., Karasawa K., Fujita Y., Sakai M. Development and Application Examples of General-Purpose/Small Water Level Gauges. Proceedings of the 2024 IEEE 13th Global Conference on Consumer Electronics (GCCE).

[8] Liu Y., Wang H., Lei X. (2021). Real-time forecasting of river water level in urban based on radar rainfall: A case study in Fuzhou City. J. Hydrol..

[9] Dhote P.R., Agarwal A., Singhal G., Calmant S., Thakur P.K., Oubanas H., Paris A., Singh R.P. (2024). River Water Level and Water Surface Slope Measurement From Spaceborne Radar and LiDAR Altimetry: Evaluation and Implications for Hydrological Studies in the Ganga River. IEEE J. Sel. Top. Appl. Earth Obs. Remote Sens..

[10] Frappart F., Blarel F., Fayad I., Bergé-Nguyen M., Crétaux J.-F., Shu S., Schregenberger J., Baghdadi N. (2021). Evaluation of the Performances of Radar and Lidar Altimetry Missions for Water Level Retrievals in Mountainous Environment: The Case of the Swiss Lakes. Remote Sens..

[11] Jan F., Min-Allah N., Düştegör D. (2021). IoT based smart water quality monitoring: Recent techniques, trends and challenges for domestic applications. Water.

[12] Kuo L.-C., Tai C.-C. (2022). Robust image-based water-level estimation using single-camera monitoring. IEEE Trans. Instrum. Meas..

[13] Sun H., Wu G., Wang X., Zhang T., Zhang P., Chen W., Zhu Q. (2022). Research on a Measurement Method for the Ocean Wave Field Based on Stereo Vision. Appl. Sci..

[14] Li D., Xiao L., Wei H., Li J., Liu M. (2022). Spatial-temporal measurement of waves in laboratory based on binocular stereo vision and image processing. Coast. Eng..

[15] Liu C., Bao H., Lan H., Yan C., Li C., Liu S. (2024). Failure evaluation and control factor analysis of slope block instability along traffic corridor in Southeastern Tibet. J. Mt. Sci..

[16] Bao H., Rao Z., Lan H., Yan C., Liu C., Liu S. (2025). Discrete element modeling method for anisotropic mechanical behavior of biotite quartz schist based on mineral identification technology. Bull. Eng. Geol. Environ..

[17] Lu Y., Liu W., Zhang Y., Li J., Luo W., Zhang Y., Xing H., Zhang L. (2021). An error analysis and optimization method for combined measurement with binocular vision. Chin. J. Aeronaut..

[18] Rahim R., Ahmar A.S., Ardyanti A.P., Nofriansyah D. (2017). Visual Approach of Searching Process using Boyer-Moore Algorithm. J. Phys. Conf. Ser..

[19] Hirschmuller H. (2007). Stereo processing by semiglobal matching and mutual information. IEEE Trans. Pattern Anal. Mach. Intell..

[20] Hou Y., Liu C., An B., Liu Y. (2021). Stereo matching algorithm based on improved Census transform and texture filtering. Optik.

[21] Yang L., Li Y., Li X., Meng Z., Luo H. (2022). Efficient plane extraction using normal estimation and RANSAC from 3D point cloud. Comput. Stand. Interfaces.

[22] Chen H., Liang M., Liu W., Wang W., Liu P.X. (2022). An approach to boundary detection for 3D point clouds based on DBSCAN clustering. Pattern Recognit..

[23] Lee S., Lim H., Myung H. Patchwork++: Fast and robust ground segmentation solving partial under-segmentation using 3D point cloud. Proceedings of the 2022 IEEE/RSJ International Conference on Intelligent Robots and Systems (IROS).

[24] Fotsing C., Menadjou N., Bobda C. (2021). Iterative closest point for accurate plane detection in unorganized point clouds. Autom. Constr..

[25] Mayer N., Ilg E., Hausser P., Fischer P., Cremers D., Dosovitskiy A., Brox T. A large dataset to train convolutional networks for disparity, optical flow, and scene flow estimation. Proceedings of the IEEE Conference on Computer Vision and Pattern Recognition.

[26] Geiger A., Lenz P., Stiller C., Urtasun R. (2013). Vision meets robotics: The kitti dataset. Int. J. Robot. Res..

[27] Bao W., Wang W., Xu Y., Guo Y., Hong S., Zhang X. (2020). InStereo2K: A large real dataset for stereo matching in indoor scenes. Sci. China Inf. Sci..

[28] Scharstein D., Hirschmuller H., Kiajima Y., Krathwohi G., Nesic N., Wang X., Westling P. High-resolution stereo datasets with subpixel-accurate ground truth. Proceedings of the Pattern Recognition: 36th German Conference, GCPR 2014.

[29] Zheng J., Peng W., Wang Y., Zhai B. (2021). Accelerated RANSAC for accurate image registration in aerial video surveillance. IEEE Access.

[30] Xiang L., Ding Y., Wei Z., Zhang H., Li Z. (2021). Research on the detection method of tunnel surface flatness based on point cloud data. Symmetry.

[31] Shi P., Yan S., Xiao Y., Liu X., Zhang Y., Li J. (2024). RANSAC back to SOTA: A two-stage consensus filtering for real-time 3D registration. IEEE Robot. Autom. Lett..

[32] Hartley R., Zisserman A. (2003). Multiple View Geometry in Computer Vision.

[33] Abdelsalam A., Mansour M., Porras J., Happonen A. (2024). Depth accuracy analysis of the ZED 2i Stereo Camera in an indoor Environment. Robot. Auton. Syst..

[34] Zhang Z. (2000). A flexible new technique for camera calibration. IEEE Trans. Pattern Anal. Mach. Intell..

